# Modeling the effect of static stretching and strengthening exercise in lengthened position on balance in low back pain subject with shortened hamstring: a randomized controlled clinical trial

**DOI:** 10.1186/s12891-020-03823-z

**Published:** 2020-12-04

**Authors:** MohammadBagher Shamsi, Maryam Mirzaei, Soodeh Shahsavari, Ameneh Safari, Morteza Saeb

**Affiliations:** 1 School of Allied Medical Sciences, Kermanshah University of Medical Sciences, Kermanshah, Iran; 2 Department of Health Information Management, School of Allied Medical Sciences, Kermanshah University of Medical Sciences, Kermanshah, Iran; 3 Esfarayen Facualty of Medical Sciences, Esfarayen, Iran; 4 Assistant professor of Orthopedic Surgery, Kermanshah University of Medical Sciences, Kermanshah, Iran

**Keywords:** Low back pain, Hamstring muscle, Y-balance test

## Abstract

**Background:**

Hamstring shortening may have negative impacts on function and biomechanics of knee and hip joints and lumbo-pelvic rhythm. Many interventions are believed to correct hamstring to its normal length. There are several reports of impairment in postural control of patients with low back pain. The purpose of this study was to compare the effect of stretching exercise and strengthening exercise in lengthened position of the hamstring muscle on improving the dynamic balance of the person in patients with chronic low back pain with short hamstring muscles.

**Methods:**

Forty-five patients with hamstring shortening who referred to physiotherapy clinic of Kermanshah university of Medical Sciences, Kermanshah, Iran were randomly allocated to the three groups; static stretching (*n* = 15), strengthening exercise in lengthened hamstring position (n = 15) and control (n = 15).

All groups received conventional physiotherapy for low back pain and the two intervention groups received stretching exercise and strengthening exercise in lengthened position programs as well. All groups performed three treatment sessions for a week, a total of 12 sessions. For balance assessment, Y-Balance test was performed for each participant in three reach directions. To determine the important and significant variables, all variables entered a model (Generalized Estimation Equations method).

**Results:**

The results indicate that based on GEE model, by controlling other variables, participants of static stretching exercise showed more improvement in balance than control group (β = 9.58, *p*-value = 0.014). Also, balance status showed significant improvement in the end of study compared to baseline of the study (β = 7.71, *P*-value< 0.001).

In addition, the balance in three reach directions improved significantly and the greatest balance improvement was in the anterior reach direction (β ranged over = 6.16 to 11.59) and the height of patients affected their balance (β = 0.28, *P*-value = 0.034).

**Conclusions:**

Group (type of intervention), phase of intervention, reach direction of test (anterior, posteromedial and posterolateral) and height of participants were associated with balance performance. Static stretching exercise was more effective than muscle strengthening exercise in lengthened position for improving dynamic balance in low back pain patients with hamstring tightness.

**Trial registration:**

Iranian Registry of Clinical Trials (IRCT201507258035n2). Registered 16th September 2015.

## Background

The hamstring muscles which are hip extensor and knee flexor have three portions including: biceps femoris, semitendinosus and semimembranosus [1]. As in most of activities such as walking, running, and swimming these muscles are active, it is necessary to keep them healthy and in normal length. Keeping knees in flexed position in many usual activities may cause shortening of hamstring muscles. Aging and inactivity assist this change [2]. Shortening of hamstring muscles may have negative impacts on function and biomechanics of knee and hip joints and lumbo-pelvic rhythm and may tend to dysfunction which may cause low back pain (LBP) [3]. Tightened hamstring increases posterior pelvic tilt and reduces lumbar lordosis, that can cause a flat back and tend to LBP [4]. Hamstring shortening may cause reduction in muscle strength, quadriceps muscle dysfunction and posture derangement leading to hyper-lordosis [5]. Many interventions are believed to correct hamstring to its normal length. This include stretching (different technique types), Mulligan’s traction straight leg raise, muscle energy technique, ultrasound therapy and short wave diathermy along with stretching exercises [5].

A common method for improving muscle tightness is static stretching [6]. One type of stretching that is applied usually is slight stretching a muscle while keeping the joint in its end-range of position known as static stretching (SS) [7].

Another approach which could be done to correct muscle length is strengthening exercise; In response to strengthening exercise, the length in which the muscle is contracted is important [8]. There is a belief that muscle contraction in its lengthened position may be useful to cause structural changes in that muscle in the form of increases in sarcomeres in series. It is thought that this type of exercise causes a long lasting effect [8]. However, studies indicating structural changes in muscles due to contraction in its lengthened position are mostly limited to animal models [9].

Obviously, prevention is the first line of health maintenance and postural stability is an important issue in harm prevention and safe functioning of the individual [2]. It is believed that balance is important for the prevention of injury and occurrence of the chronic LBP [10, 11].

Maintaining the entire body’s balance is a complex action, requiring interaction between different body systems. There are several reports of impairment in postural control of patients with low back pain [3, 5, 7, 12–15] which point out the increased postural instability in patients with low back pain due to the disorders of lumbar and spinal muscle strength, coordination, and coupling and, finally, decreased diversity of strategies controlling postural stability [16].

Given the fact that the lumbar postural changes in different daily activities can change the center of gravity, it can affect postural stability [17].

Many studies suggest that chronic LBP might cause an alteration in motor control because of pain inhibitory mechanisms [18, 19]. LBP prevents activation of trunk muscles, affects spinal stabilization system, and leads to impaired postural correction strategies. This inhibition due to back pain makes some challenges to trunk stabilization [20].

Apparently, considering the spine alignment, especially the lumbar spine, during daily activities, studying the effect of changes in lumbar curvature on postural stability is of great importance. Since hamstring muscle has a definite effect on pelvic tilt and lumbar spine curvature, shortness of this muscle can have indirect effects on postural stability [12–17].

Dynamic balance is measured by the Y Balance Test (YBT) which is simple and yet reliable. In order to standardize the modified Star Excursion Balance Test (mSEBT), this test was introduced. It is more practical and commercially available and so due to its simplicity and reliability, it has become an extremely popular test [12].

The YBT, has been reported to be a valid and reliable measure of dynamic balance; furthermore, the results of the YBT have been reported to be related to lower-extremity impairments and to be predictors of injuries [13].

The purpose of this study was to compare the effect of stretching and strength exercises of the hamstring muscle length on improving the dynamic balance of the person, using Y test in patients with chronic LBP with short hamstring muscles.

## Methods

### Study design and settings

This study was part of a parallel group, randomized-controlled trial that run from July 2016 through March 2017 (registration no. IRCT201507258035n2 in the Iranian Registry of Clinical Trials) [21]. Eligible subjects were recruited from the physiotherapy clinic of Kermanshah University of Medical Science, Kermanshah, Iran. To conduct the research, we received permission from the Ethic Committee of Kermanshah University of Medical Science (code number: kums.rec.1395.169).

### Participants

In the first session, the study was explained to all participants; they read and filled out the informed consent form about the study purpose and procedure. Three consecutive or 5 intermittent absences in attending sessions, caused them to be excluded. Using the Pocock formula with considering of 80% power and 95% confidence level, the sample size was computed. Regarding the results of a previous clinical trial and based on the change in the mean score of hamstring flexibility (Mean_1_ = 5.7, Mean_2_ = 3, SD_1_ = 3.33, SD_2_ = 2.9) [22], 15 patients were estimated per group.

**Fig. 1:**
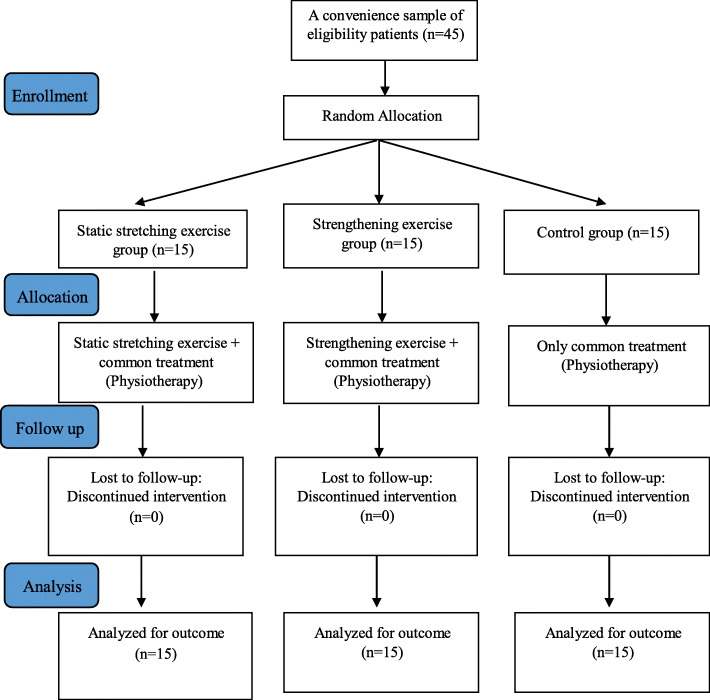
Summary of patient’s flow diagram

After receiving all the permissions and approvals, then, forty-five eligible subjects were randomly assigned to three groups of static stretching (*n* = 15), strengthening exercise in muscle lengthened position (n = 15) and control (n = 15), using a block randomization procedure of size 2 (Fig. 1). In this clinical trial, random allocation to each group was performed by a statistician. This person who defined allocation sequences for the study, those who assigned patients to each group and the evaluator were blinded to the patients’ allocation sequence and the allocation sequence remain concealed until patients were enrolled and assigned to interventions.

Study eligibility criteria included having non-specific LBP for more than 3 months, pain intensity from 3 to 6 according to the visual analogue scale (VAS), obvious hamstring muscle shortness in SLR test, and age between 18 and 60 years. To perform the SLR test, the participants were positioned in supine with fewer clothes and without a pillow under their head, their hips medially rotated and adducted, and their knees extended. The tester lifted the patient’s legs forcing the posterior ankle upward while keeping their knees in a fully extended position. The tester continued to lift the participant’s legs by flexing at the hips until they complained of pain or tightness in their backs or back of their legs [23]. Having any pathology or anomaly in lower limbs such as neuropathic pain, malignancy, inflammatory diseases, and severe osteoporosis, arthritis and/or bone diseases were the study exclusion criteria. The patients and researcher were not aware of the existence of different intervention groups.

### Intervention

Common conventional physiotherapy program for all three groups included 15 min of heat therapy (hot pack), 15 min application of transcutaneous electrical nerve stimulation (High frequency TENS) to the area of low back and performing the commonly used exercises for back pain. While the control group received only routine above care, subjects in the exercise groups received additional interventions.

In the first group, passive static stretching (SS) of the hamstring was performed using a spring for three sets of 2 min with 2 min rest in between while the subjects were in supine position and knee was fully extended. In the second intervention group or strengthening in lengthened position (SLP) group, in seated posture on a chair, while the thighs of subjects were supported on a surface and their hip joints were kept in 120 degrees of flexion (knee joint in full extension); subjects extended their hip against a spring and contracted hamstring muscles. The other hip and knee joints were kept flexed in 90 degrees. In the control group, nothing other than the common interventions (heat therapy and TENS) were done for the participants. The interventions were applied during 12 sessions, three times per week.

### Outcome measure

#### Assessment of balance

YBT is one of the most important tests for the evaluation of dynamic balance. This is a functional test, which requires strength, flexibility, and neuromuscular control, and stability, range of movement, balance, and proprioception. Because of its speed, efficiency, portability, consistency, and objectivity, it is a popular functional test [11].

The YBT needs lower limb strength and range of motion [12]. The advantages of this test are its standard protocol and high inter-rater (0.99–1.00) and intra-rater (0.85–0.91) reliabilities [13]. The reliability of YBT is reported to be for the anterior 0.99; posteromedial 1.0; posterolateral 0.99; and composite 0.97 [16].

For balance assessment, Y-Balance test was performed in three directions (anterior (ANT), posteromedial (PM) and posterolateral (PL)) for all participants at the baseline and after 12 sessions of intervention.

In this test, the participants tried to reach the anterior, posteromedial, and posterolateral reach directions with one foot as far as they were able while they were standing on the other foot. If their reaching foot kicked the ground or the stance foot failed to maintain a unilateral stance on the ground, the test was recognized as invalid. The maximum reach distance of three trials in each reach direction was recorded by investigator and the mean value in each reach direction was chosen for data analysis. The test was done for both limbs. The reach distance data were normalized to lower limb length. For lower limb length, while lying in the supine position, the distance between anterosuperior iliac spine (ASIS) and the center of the ipsilateral medial malleolus of tibia was measured.

#### Predictor variables

To determine the important and significant variables, all variables, including age, sex, height, weight, leg dominance (right or left), reach direction, phase (before and after intervention), type of intervention (three study groups), pain and disability entered the model and the significant variables were entered the final model. Visual Analogue Scale (VAS) (0–100; 0 – no pain; 100 – worst pain you can imagine) and Persian translated version of the Oswestry disability questionnaire (0 = no disability, 100 = totally disabled) were used to assessment of pain intensity and functional status, respectively [24].

### Statistical analyses

Quantitative and qualitative data were expressed as mean (SD) and frequency (percent), respectively. The possible differences in demographic and clinical features between groups were evaluated using the analysis of variance (ANOVA) and chi -square test. The normality of data distribution was confirmed (Kolmogorov-Smirnov test).

The Generalized Estimation Equations (GEE) method was used to analyze variation in outcomes across the intervention groups and predictor variables. GEE are methods of parameter estimation for correlated data. When data is recorded with a measurement unit in a time sequence, these repeated measurements are correlated. If these conditions are not considered, the standard errors of the parameter estimates will be large and the significantly results will be not reliable.

So, considering the structure of correlations between observations, GEE was run to investigate the adjusting effect of variables on Y-Balance test reach directions (dependent variables). By the way, unlike general linear models that applied to the independent measures for each subject, this model is able to identify patterns for subjects with correlated measures, (12 measurement of Y-Balance test).

In the first step, a backward strategy to select significant variables was applied at *P*-value< 0.05, then the significant variables were entered in the final model.

Analyze of the experimental data was started through the use of  SPSS ver.22 (SPSS Inc., Chicago, IL, USA) software and two-sided *P*-values < 0.05 were considered as level of significant.

## Results

In 49 patients, 15 (31%) was women. The mean value of age was 37.73(SD = 11.4) years. The demographic characteristics of the study participants is summarized in Table 1. There was no significant difference in any of these characteristics (age, weight, height, gender, pain and disability) in the three groups (*P* > 0.05).

**Table 1:** Baseline characteristics of the LBP patients in each group

groups	Static Stretch (***n*** = 15)	Strengthening Exercise (n = 15)	Control (n = 15)	*P*-value
**Age (year)**	37.67 (8.96)	37.07 (13.39)	39.12 (11.61)	0.823^**#**^
**Height (cm)**	171.93 (13.21)	172.64 (10.14)	172.31 (10.14)	0.995^**#**^
**Weight (kg)**	76.57 (13.05)	81.54 (16.59)	80.91 (14.1)	0.883^**#**^
**BMI (kg/m**^**2**^**)**	25.90 (3.15)	26.82 (4.21)	27.47 (3.17)	0.535^**#**^
**VAS**	27.2 (24.51)	35.77 (18.91)	33.71 (23.11)	0.572
**Disability**	24.95 (13.84)	23.45 (13.53)	16.17 (10.67)	0.211
**Sex**				
**Female**	5 (33.3)	4 (26.7)	5 (33.3)	0.726^*^
**Male**	10 (66.7)	11 (73.3)	10 (66.7)	

*LBP* Low Back Pain, *BMI* Body Mass Index, *VAS* Visual analogue scale. Data are means (SD) except sex that presented as number (percent)/ ^**#**^Based on on-way ANOVA test/ ^*^Based on chi-square test

**Fig. 2:**
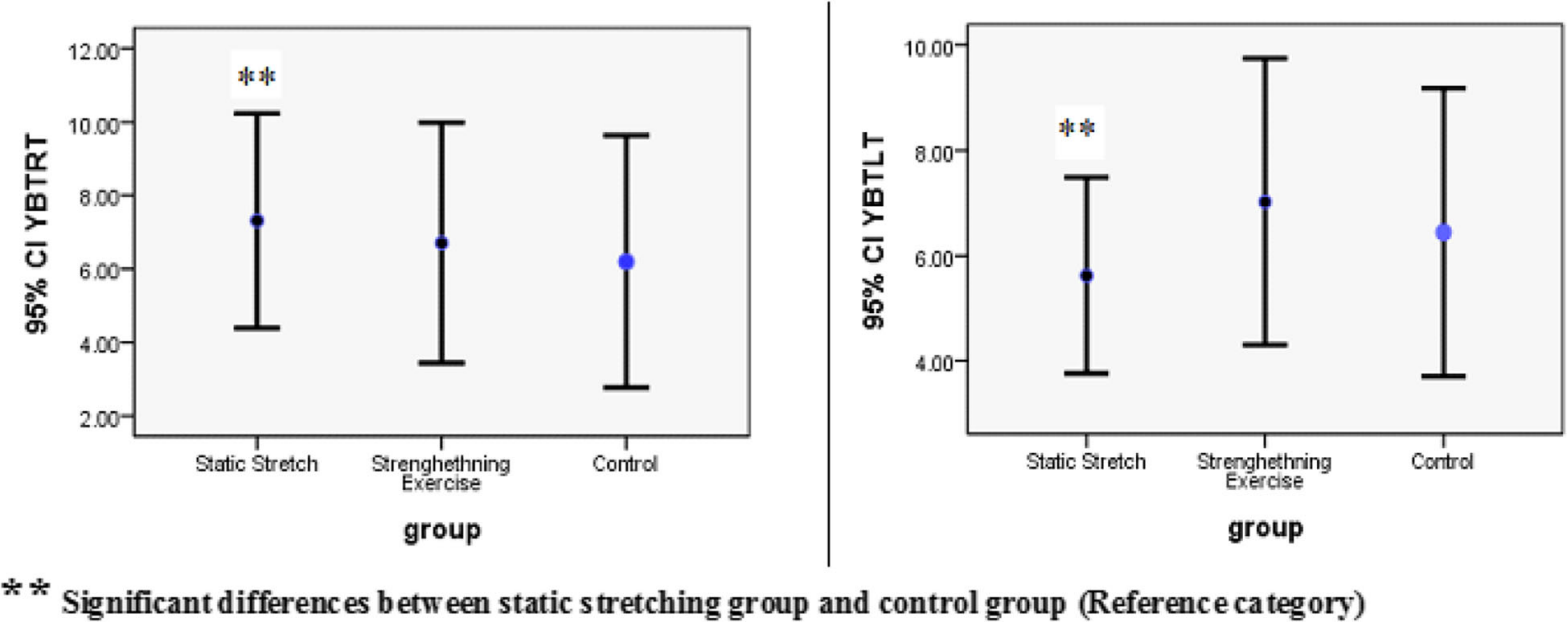
Error bar plots for the mean differences of balanced measures/ (RtAnt: Right Anterior, RtPL: Right posterolateral, RtPM: Right posteromedial, (LtAnt: Left Anterior), LtPL: Left posterolateral, LtPM: Left posteromedial)

Figure 2 shows the results of balance test before and after the interventions and for different reach directions among the three groups, using error bar graph. As shown, in all groups and in all reach directions, mean values of balance test measured before and after the interventions, have increased significantly. It is also observed that the improve in balance values were similar between the left and right lower limbs, however different among ANT, PL and PM reach directions in both limbs; the greatest balance improve was observed in the anterior reach direction. The mean and standard deviation of the balance test values are shown in Table 2.

**Table 2:** Mean and SD for the Balanced Measures in three groups

Position	Phase	Static stretching	Strengthening	Control
		Mean (SD)	Mean (SD)	Mean (SD)
Right ANT	Pre	81.27 (13.77)	81.33 (10.06)	79.27 (9.16)
	Post	85.60 (12.24)	84.20 (11.69)	84.0 (11.16)
	MD (95% CI)	4.33 (0.49 to 8.17)	2.86 (− 0.42 to 6.15)	4.73 (− 0.38 to 9.85)
Right PL	Pre	77.40 (16.48)	77.13 (9.41)	70.40 (16.46)
	Post	85.80 (14.91)	85.07 (14.18)	77.53 (15.49)
	MD (95% CI)	8.40 (0.96 to 15.83)	7.93 (0.17 to 15.69)	7.13 (1.5 to 12.73)
Right PM	Pre	72.93 (17.37)	70.53 (16.38)	67.40 (12.87)
	Post	82.13 (15.20)	79.87 (16.83)	74.13 (15.75)
	MD (95% CI)	9.20 (5.11 to 13.28)	9.33 (3.31 to 15.35)	6.73(−1.5 to 14.94)
Left ANT	Pre	81.53 (13.30)	79.47 (12.56)	79.80 (7.38)
	Post	86.00 (13.55)	83.27 (13.58)	85.13 (10.16)
	MD (95% CI)	4.46 (1.54 to 7.38)	3.80 (0.62 to 6.97)	5.33 (0.51 to 10.15)
Left PL	Pre	80.20 (15.95)	77.53 (17.23)	75.33 (13.14)
	Post	86.00 (15.78)	86.26 (17.16)	81.00 (15.33)
	MD (95% CI)	5.80 (2.54 to 9.05)	8.73 (2.68 to 14.73)	5.66 (0.56 to 10.76)
Left PM	Pre	71.27 (17.04)	69.00 (17.84)	67.33 (13.65)
	Post	77.87 (16.65)	77.53 (16.41)	75.66 (17.41)
	MD (95% CI)	6.60 (2.44 to 10.75)	8.53 (3.30 to 13.76)	8.33 (5.17 to 10.46)

Mean (SD) was reported/ MD (95% CI): Mean difference and 95% confidence interval

To assess the balance state in different groups and different situations, GEE model was fitted. The results of this model are shown in Table 3.

**Table 3:** Multivariate analysis of balance test bases on GEE model

Characteristics		β	SD(β)	95%CI	*P*-value ^b^
Group	Static stretching	9.58	3.90	(1.93,17.22)	0.014
	Strengthening	0.273	3.61	(−7.35,6.80)	0.94
	Control	Reference	–	–	–
Phase	post	7.71	1.18	(5.39,10.02)	< 0.001
	pre	Reference	–	–	–
Reach direction	Right ANT	11.59	1.69	(8.27,14.93)	< 0.001
	Right PM	6.16	1.38	(3.43,8.88)	< 0.001
	Right PL	Reference	–	–	–
	Left ANT	11.28	1.78	(7.79,14.77)	< 0.001
	Left PM	9.33	0.80	(7.75,10.91)	< 0.001
	Left PL	Reference	–	–	–
Height		0.28	0.13	(0.021,0.538)	0.034

^a^ Reference category; ^b^ Based on adjusted model; dependent variable = balance test

Based on univariate model, the type of intervention, time, reach direction, and height were significant and so these variables were used in the final model.

The results indicate that based on GEE model, by controlling other variables, participants of static stretching exercise showed more improvement in balance than other groups (β = 9.58, *p* = 0.014), and mean balance value in this group was 9-folds more than the control group. However, the strength training group had no significant difference in terms of balance status with the control group (β = 0.27, *p* = 0.94). The results also showed that in all three groups, the balance in the ANT, PL and PM reach directions improved significantly (*P* < 0.001) and the greatest balance improvement was in the anterior reach direction(β ranged over = 6.16 to 11.59).

The height of individuals affected their balance and the level of balance increased significantly with increasing height (β = 0.28, *p* > 0.05) (Table 3).

## Discussion

This clinical trial study was conducted to compare the effect of static stretching and strengthening exercises in lengthened position on the dynamic balance of patients with chronic low back pain. The results of this study showed that group (type of intervention), phase of intervention, reach direction of balance test (ANT, PL and PM) and height of participants were associated with balance performance. In the static stretching exercise group, balance improved more than other groups. However, improvement in balance was not different in strength training group from the control one.

The ability of the body to be stable in static and dynamic situations is called postural stability. In daily activities and sport exercises having dynamic balance for maintaining stability during different tasks performance is essential [14]. For the daily activities, we need a healthy balance control system, muscular strength, and flexibility [25].

In a study to investigate the effect of hamstring and quadriceps muscle strength on dynamic and static balance of athletes, it was found that increasing quadriceps muscle strength improves the balance. However, hamstring muscle strength did not affect the balance [26]. It is in line with our study that increasing hamstring muscle strength has not a prominent effect on the balance. In another study, to assess the relationship between the ratio of hamstring to quadriceps muscle strength and dynamic balance, it was shown that this ratio was related to the dynamic body balance in healthy subjects, but not in patients with knee cruciate ligament injury [27]. Meanwhile, in a similar study performed on patients after cruciate ligament surgery, there was a positive correlation between knee strength and dynamic balance. In this study, the strength of hamstring muscles was more closely related to the dynamic balance, according to Y balance test, in three reach directions [28]. In a study on hamstring muscle shortness, there was a significant negative correlation between muscle shortness and dynamic balance [29]. It is in line with our results that improving hamstring shortness in SS group caused better balance.

There are more documents on hamstring flexibility and balance. In a study conducted by Yalfani et al. (2017) on patients with chronic low back pain, after 6 weeks of aquatic exercise, the patients’ balance (assessed by Y balance test) and their hamstring flexibility improved significantly. It may be possible to attribute their balance improvement to increasing hamstring muscle flexibility [30]. The effect of yoga on balance and flexibility of hamstring using forward reach and sit and reach tests on patients with chronic low back pain was investigated in a pilot study by Galantino et al. Balance and flexibility improved and disability decreased in the yoga group. The improvement in balance is associated with increased hamstring flexibility in this study too [31].

Static stretching has been defined as a tool for elongating the muscle to tolerance and sustaining the position for a length of time. Static stretching is a muscle stretch resulting in placement of the muscle in its long state and maintaining this condition for some time that can resolve muscle shortness. Some authors consider a greater effect of static stretching on the flexibility of hamstring muscle than active exercise methods [32, 33].

As hamstring contraction causes the knee joint movement, stretching exercises of this muscle increases the range of motion of this joint. It is believed that passive hamstring stretching exercises may increase the range of motion of distant joints. That is because the force of stretching is transmitted to other joint by the fascias that connect human skeletal muscles with each other. So it stabilizes the pelvic and spinal muscles, and therefore improves balance [34].

Perhaps the higher effect of static stretching on improving dynamic balance maybe due to the greater effect of this type of intervention on reducing hamstring muscles’ tightness and increasing their flexibility.

The greatest balance improve was observed in the anterior reach direction. Some studies indicate more sensitivity and specificity of the scores of anterior reach direction than composite scores to identify individuals as at risk [35, 36]. The explanation for more improvement in anterior direction may be using visual feedback in anterior reach direction compared to other directions [16, 36]. It is clear that postural-control strategies are used while performing the test and influence reach extent. The visual system makes body orientation in space using visual cues. The somatosensory and visual subsystems are the primary bases of balance and postural awareness [16].

The height of participants was associated with balance performance. Normalizing reach scores to length of lower limb is for eliminating personal differences between participants. Therefore, it seems usual that balance performance is different regarding height of patients.

Important strengths of this study was having a control group in addition to two intervention ones. The clinical implications of this study could be application of static stretching exercise for shortened hamstring muscles in LBP patients.

Current study had some limitations: Some participants had difficulty in performing YBT due to other problems for doing one-leg balance. Because of the present study was part of another clinical trial with variables related to EMG activity of shortened hamstring muscles, sample size was estimated based on a changes in hamstring flexibility. To produce more satisfactory and generalized results, more studies on change in balance, as a primary outcome, is recommended. Different existed factors may possibly affect the balance, therefore, other possible influencing factors should be sought and investigated.

## Conclusions

In conclusion, based on the results of this study, group (type of intervention), phase of intervention, reach direction of test (anterior, posteromedial and posterolateral) and height of participants were associated with balance performance. Static stretching exercise was more effective than muscle strengthening exercise in lengthened position for improving dynamic balance in LBP patients with hamstring tightness.

## Data Availability

The datasets used and/or analyzed during the current study are available from the corresponding author on reasonable request.

## Abbreviations

ANT: Anterior
LBP: Low back pain
GEE: Generalized estimating equation
PM: Posteromedial
PL: Posterolateral
SLP: Strengthening in lengthened position
SS: Static stretching
VAS: Visual Analogue Scale
YBT: Y Balance Test
